# Supplementary material for: GALA: a computational framework for de novo chromosome-by-chromosome assembly with long reads
Source: Nat Commun. 2023 Jan 13;14:204. doi: 10.1038/s41467-022-35670-y (PMC9839709; doi:10.1038/s41467-022-35670-y)
Supplement: Supplementary file 1 — Supplementary Information [file 41467_2022_35670_MOESM1_ESM.pdf]

Supplementary Information

GALA: a computational framework for *de novo* chromosome-by-chromosome assembly with long reads

Mohamed Awad[1] & Xiangchao Gan[1,2*]

[1]Max Planck Institute for Plant Breeding Research, Department of Comparative Development and Genetics, Carl-von-Linné-Weg 10, 50829 Köln, Germany

[2]State Key Laboratory for Crop Genetics and Germplasm Enhancement, Academy for Advanced Interdisciplinary Studies, Nanjing Agricultural University, Nanjing 210095, China

[*] Author for correspondence: e-mail address (gan@mpipz.mpg.de)

Supplementary Table 1: Metrics of preliminary assemblies and unpolished GALA assemblies for *C. elegans* and *O. sativa* genomes. Here "_Corr" referes to the preliminary assembly produced using the corrected reads.

Species	Database	Assembler	Contigs	Genome Size	N50	L50	Longest Contig
C.elegans	PacBio - all runs	Canu	214	109716008	4217904	9	9058364
		Flye_Corr	68	104652135	3693393	9	12185728
		Miniasm_Corr	134	105138292	2555369	14	5394256
		Miniasm	469	115467549	2387630	17	4834144
		Wtdbg2	356	104682976	1850992	19	5311454
		Mecat_500	86	101501420	1917460	19	5098523
	PacBio (SRR7594465)	Flye_Corr	41	102134972	4201097	9	10503810
		Wtdbg2_Corr	181	102181086	2697772	13	6773398
	Nanopore	Miniasm	127	114573559	3813016	10	9611247
		Necat_3000	73	110408870	2860026	13	8308214
	GALA	GALA assembly without manual intervention	13	102851250	9443779	4	21328098
		GALA final assembly (Un-polished)	7	102269907	17797375	3	21328098
O. sativa	Nanopore	Canu	103	384832701	17009720	8	40643309
		Flye	39	240299423	24863102	4	44036194
		Flye_Corr	112	119953657	16979905	7	3730224
		Wtdbg2	1128	375751940	4039823	26	14367081
		Wtdbg2_Corr	2166	417867573	1680652	60	11531817
		Miniasm	111	388020961	17182649	9	29543518
		Miniasm_Corr	89	382442769	17479396	9	29488258
		Necat	44	380674828	24142127	7	32076659
	GALA	GALA assembly without manual intervention	16	378304357	30733426	6	378172913
		GALA final assembly (Un-polished)	14	378172913	30733426	6	378172913

Supplementary Table 2: Comparison between the best preliminary assembly, GALA assembly without telomeric motif analysis and manual intervention, and the GALA final assembly.

Species	Chr	The best preliminary assembly				The assembly by GALA without manual intervention					**GALA final assembly**	
		Contigs	N50 (Mb)	L50	Longest contig (Mb)	Scaffolding groups	Contigs	N50 (Mb)	L50	Longest contig (Mb)	Contigs	Length
C.elegans	1	4	4.20	2	6.51	1	2	10.10	1	10.10	1	15.40
	2	4	5.24	2	5.79	1	1	15.56	1	15.56	1	15.57
	3	8	2.66	3	3.62	2	2	7.35	1	7.35	1	14.00
	4	9	4.18	2	4.21	1	3	9.05	1	9.05	1	17.80

	5	3	5.44	2	10.50	1	1	21.32	1	21.32	1	21.33
	6	8	3.34	2	5.90	1	2	9.44	1	9.44	1	18.15
O.sativa	1	2	28.30	1	28.30	1	1	44.16	1	44.16	1	44.16
	2	3	22.18	1	22.18	2	2	22.15	1	22.15	1	35.84
	3	3	24.28	1	24.28	1	1	38.15	1	38.15	1	38.15
	4	4	27.53	1	27.53	1	1	34.05	1	34.05	1	34.05
	5	3	17.39	1	17.39	1	1	30.73	1	30.73	1	30.73
	6	2	13.98	1	13.98	1	1	32.07	1	32.07	1	32.07
	7	2	15.5	1	15.5	1	1	29.47	1	29.47	1	29.47
	8	2	16.67	1	16.67	1	1	29.55	1	29.55	1	29.55
	9	2	19.77	1	19.77	1	1	23.37	1	23.37	1	23.37
	10	1	23.99	1	23.99	1	1	24.14	1	24.14	1	24.14
	11	3	10.88	2	12.52	2	2	18.11	1	18.11	1	29.46
	12	3	9.22	2	9.22	1	1	26.63	1	26.63	1	26.63

Supplementary Table 3: The number and the size of collapsed regions in preliminary assemblies and the unpolished GALA assemblies for *C. elegans* and *O. sativa* genomes.

Species	draft	Number_of_collapses	Size_of_collapses
C.elegans	Flye_corr (SRR7594465)	108	873935
	Mecat_500	117	876029
	Miniasm	127	890946
	GALA	20	170708
O.sativa	Canu	30	887159
	Necat	54	990523
	Miniasm	34	1188260
	GALA	46	690167

Supplementary Table 4. The statistics of *C. elegans* assemblies using different coverages of PacBio Sequencing data by Flye and GALA with or without Hi-C data. The statistics for gapped assemblies are shown in blue.

	Number of scaffolds				N50 of the assembly			
Coverage (X)	Flye	GALA without Hi-C	Flye/Hi-C	GALA/Hi-C	Flye	GALA without Hi-C	Flye/Hi-C	GALA/Hi-C
20	652	96	192	14	260,780	1,851,699	1,735,613	15,966,384
30	374	41	149	18	659,879	4,165,642	1,867,656	8,477,738
40	68	26	44	17	2,281,700	6,249,032	8,122,814	14,150,196
50	60	28	45	15	3,047,053	5,364,198	6,220,146	14,282,330
60	51	33	38	22	3,568,950	5,196,967	6,552,095	14,132,688
70	50	23	41	16	4,016,141	6,275,636	5,443,425	14,186,604
80	47	27	41	20	4,159,244	5,389,667	5,896,181	6,825,305
90	46	27	40	15	4,044,388	5,879,863	4,783,642	14,178,668
100	42	28	35	16	4,209,404	6,851,827	6,558,522	14,175,551

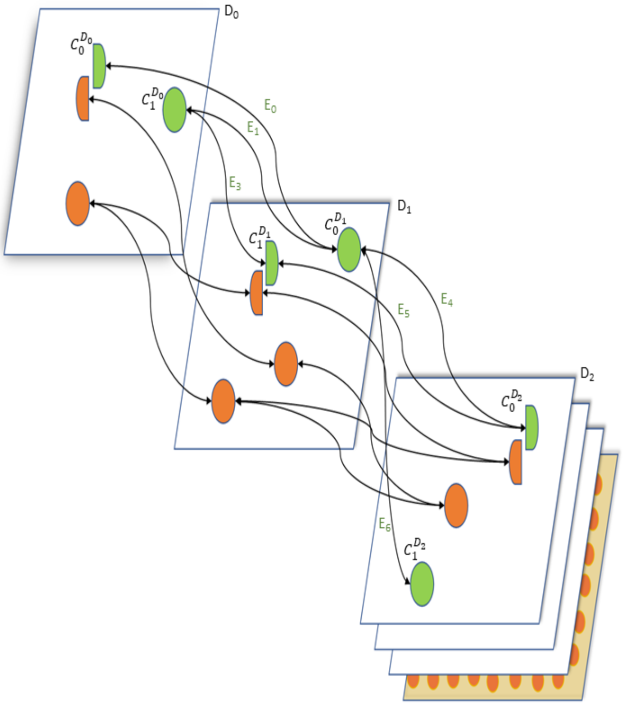

Supplementary Figure 1. An Illustration of CCM module: showed an example for building Scaffolding groups of the green contigs. The inter-layer edges E0 = {$C_0^{D_0}$, $C_0^{D_1}$ } and E1= {$C_1^{D_0}$, $C_0^{D_1}$} enable the node $C_0^{D_1}$ in D1 and nodes $C_0^{D_0}$ and $C_1^{D_0}$ in D0 to reach each other. So, ($C_0^{D_0}$ and $C_1^{D_0}$) form a single Scaffolding group of layer D0. We can also merge ($C_0^{D_0}, C_1^{D_0}, C_0^{D_1}, C_1^{D_1}, C_0^{D_2}$ and $C_1^{D_2}$) in a single Scaffolding group. Using the same approach, we can traverse through the rest of the contigs in each layer and allocate all nodes into different Scaffolding group.

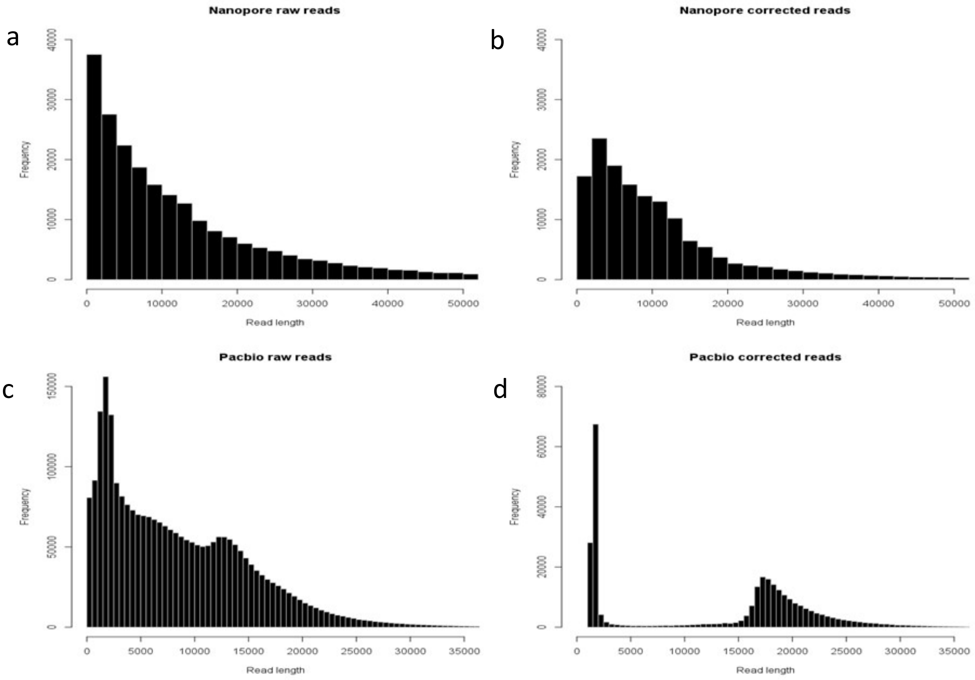

Supplementary Figure 2. The distributions of the length of the reads used for the assembly of *C. elegans* genome. (a) Nanopore raw reads. (b) The self-corrected Nanopore reads by canu [1]. (c) PacBio raw reads. (d) The self-corrected reads by canu [1].

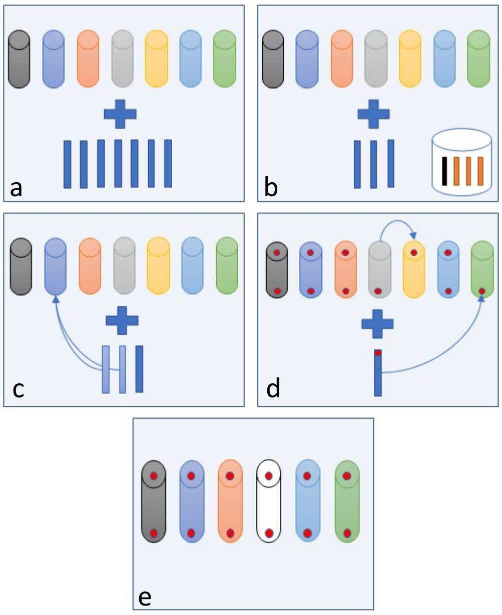

Supplementary Figure 3. The telomeric motif based analyses to merge several Scaffolding groups in *C. elegans*. (a) GALA was applied on preliminary assemblies and raw reads, and produced seven scaffolding groups and seven short continuous contigs. (b) NCBI-blast showed that three contigs are from bacterial contamination (orange), a 13 kb mitochondrial genome (black). (c) Two of the remaining contigs were anchored to one of the Scaffolding group by Miniasm/Nanopore assembly. (d) Four Scaffolding groups had telomere motif at both terminals indicating the complete chromosome; two (grey and yellow) had only one telomere and were from a single chromosome indicated by their sizes; the last group (green) had only one telomere and the missing telomere appeared on the remaining short contig. (e) The chromosome-by-chromosome assembly successfully assembled each Scaffolding group.

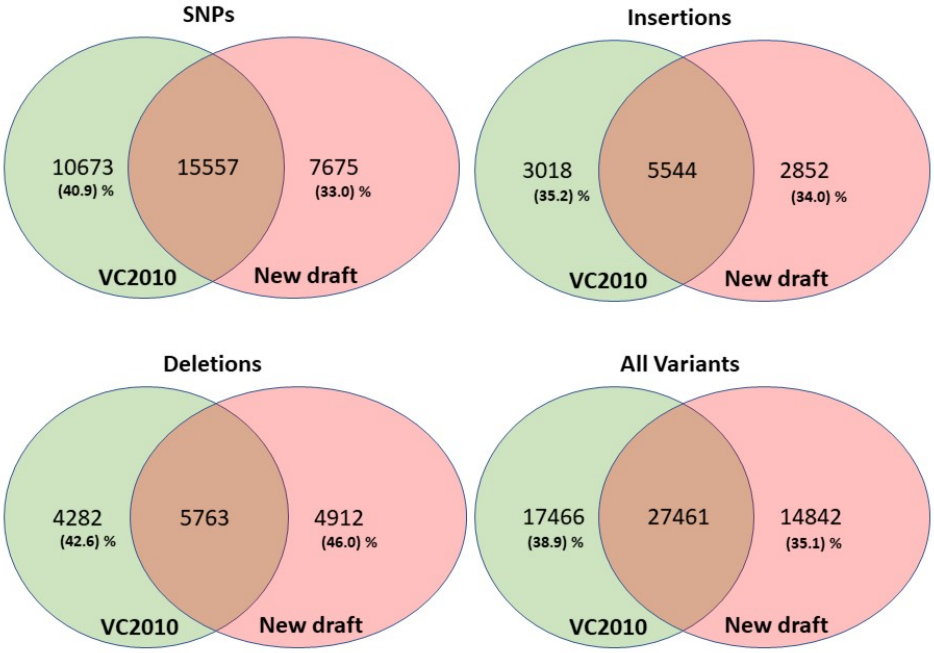

Supplementary Figure 4. Variant calling of *C.elegans* VC2010 and our new assembly against N2 reference. BWA [2] used for mapping and Denom [3] used for variant calling.

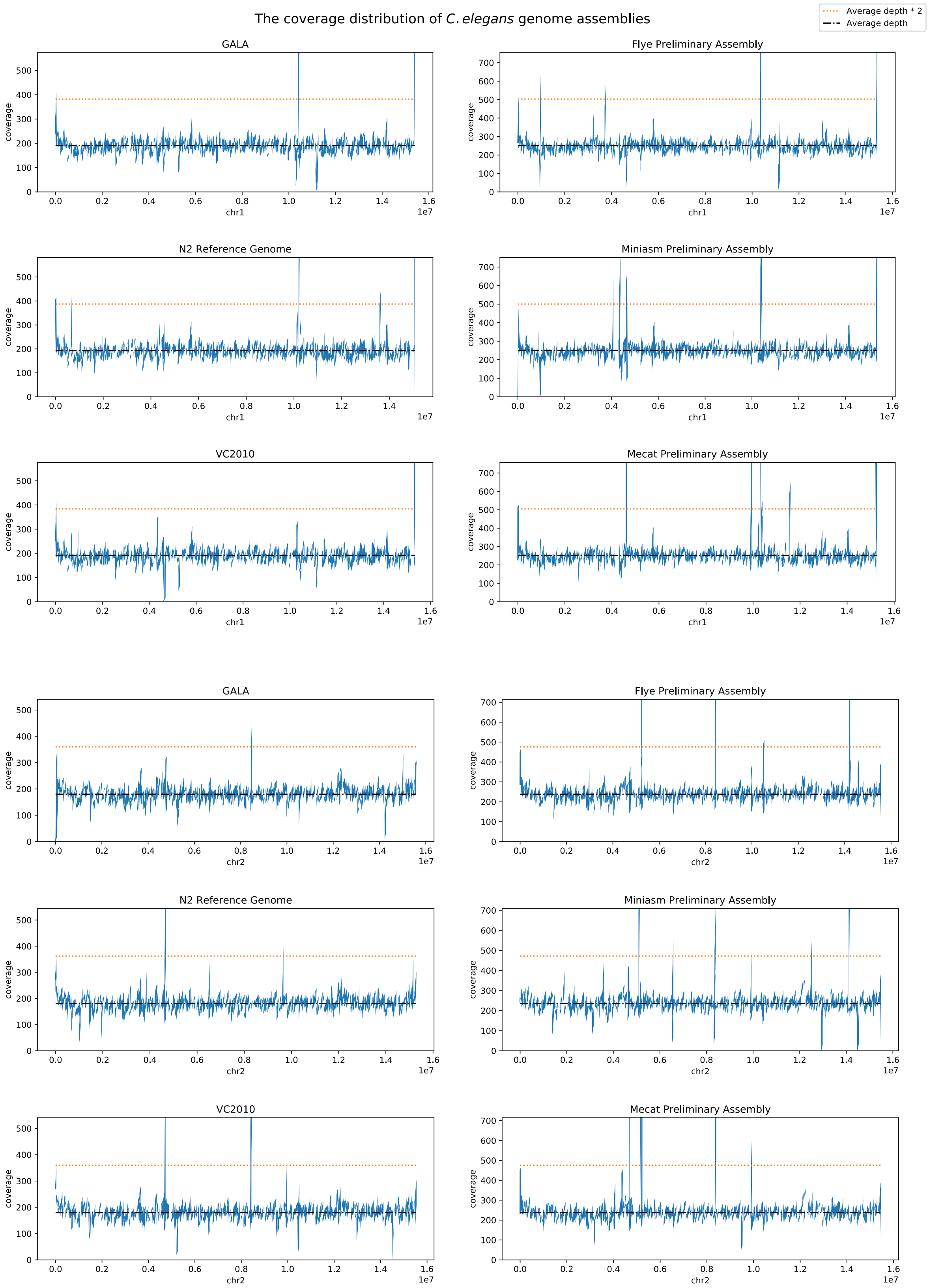

The coverage distribution of C. elegans genome assemblies
Average depth * 2
Average depth
GALA
Flye Preliminary Assembly
N2 Reference Genome
Miniasm Preliminary Assembly
VC2010
Mecat Preliminary Assembly
coverage
chr1
chr2
1e7

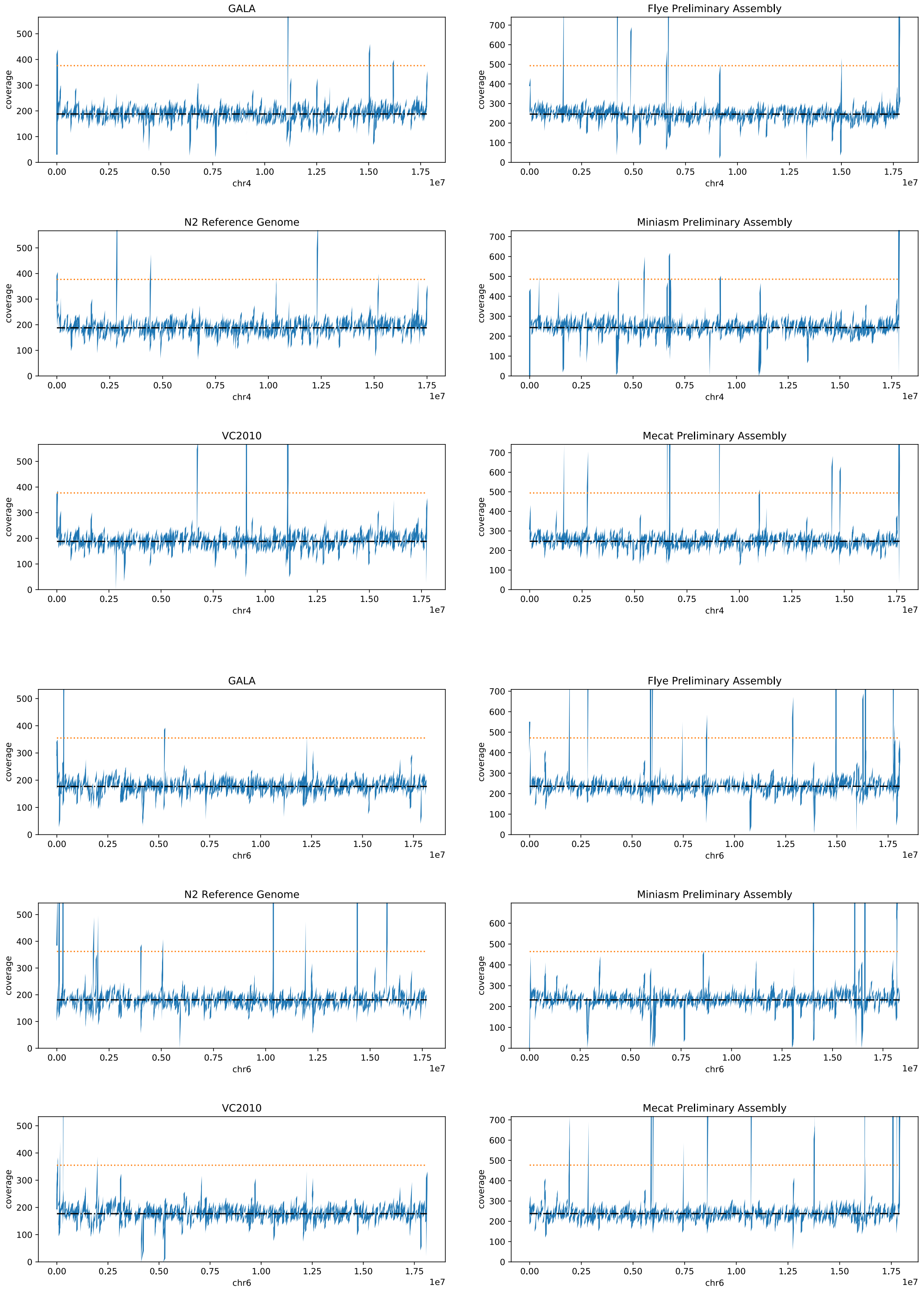

Supplementary Figure 5. Distributions of the depth-of-coverage when aligning the raw PacBio reads from *C. elegans* genome to the Gala assembly, N2 reference genome, VC2010 assembly, Flye preliminary assembly, Miniasm preliminary assembly and Mecat preliminary assembly. For simplicity, only chr1, chr2, chr4 and chr6 are shown here. The GALA assembly shows better performance than the preliminary assemblies and N2 reference genome and are comparable to the VC2010 assembly.

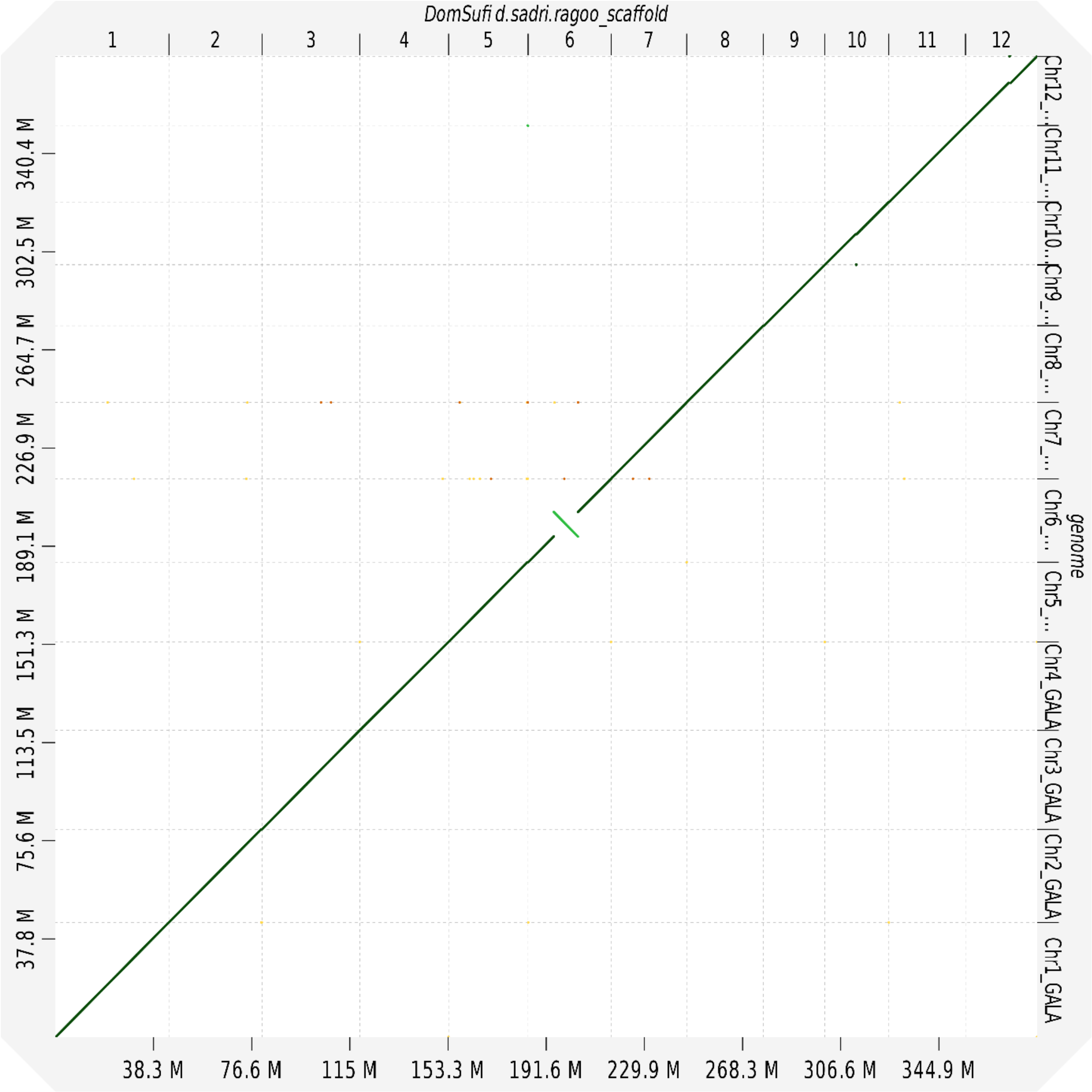

Supplementary Figure 6. The dotter plot of the alignment between our assembly of *Oryza sativa* sadri landrace and the published *reference-guided* assembly. The inversion is absent in the published assembly as the reference-guided scaffolding arranged the region with the Nipponbare reference genome which does not contain the inversion [4].

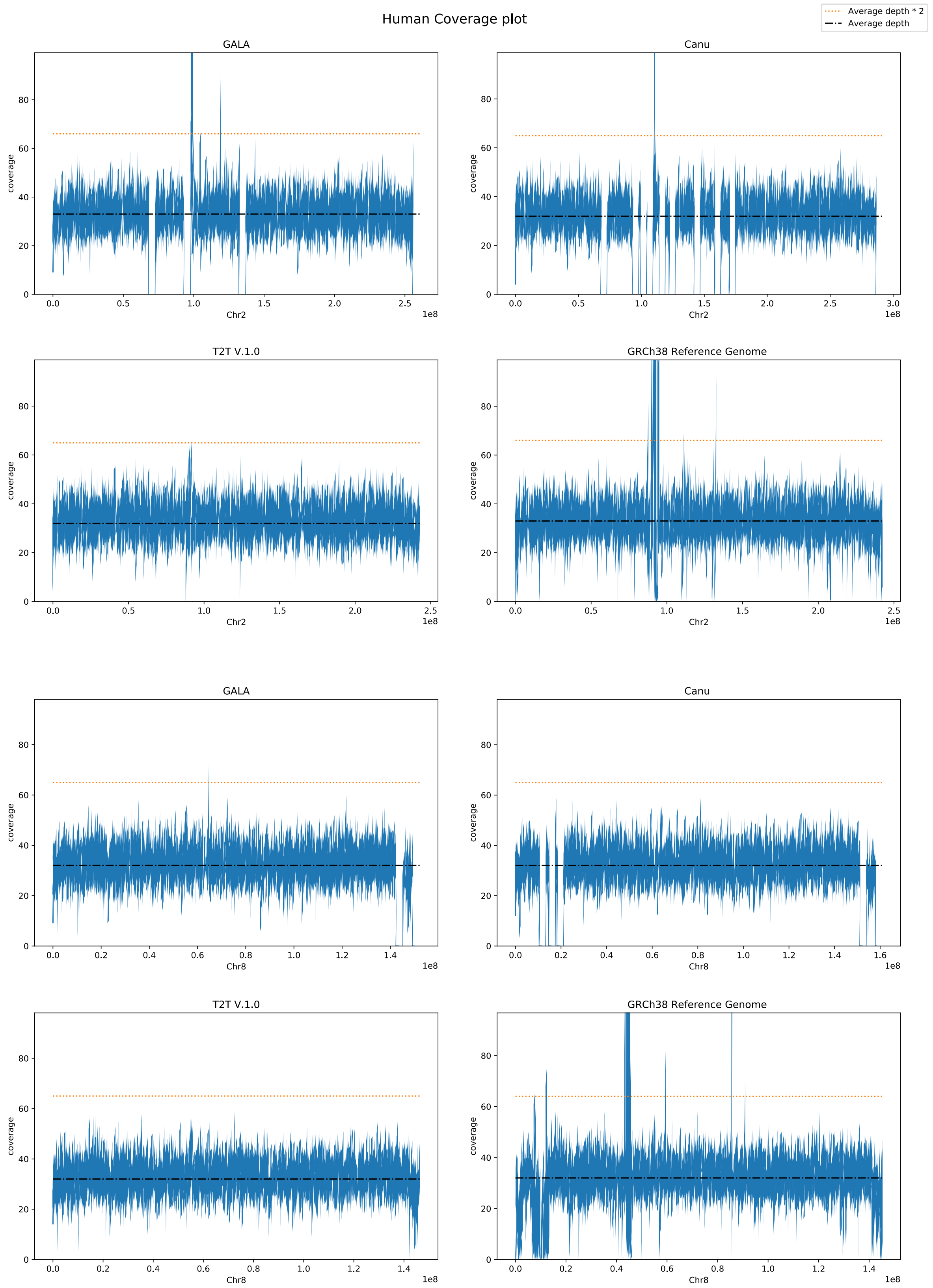
Human Coverage plot
Average depth * 2
Average depth
GALA
Canu
T2T V.1.0
GRCh38 Reference Genome
coverage
Chr2
Chr8
1e8

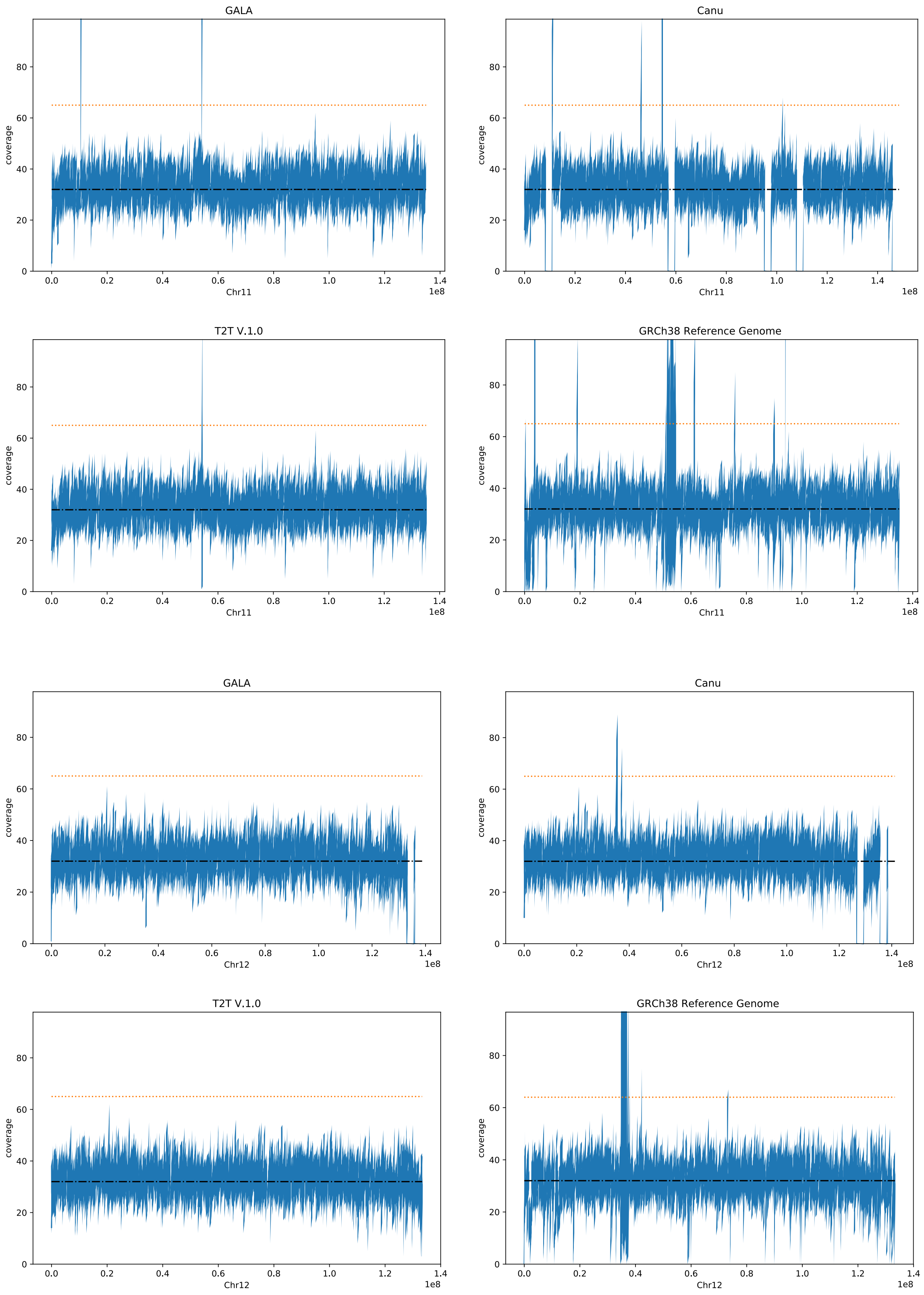
GALA
Canu
T2T V.1.0
GRCh38 Reference Genome
coverage
Chr11
Chr12
1e8

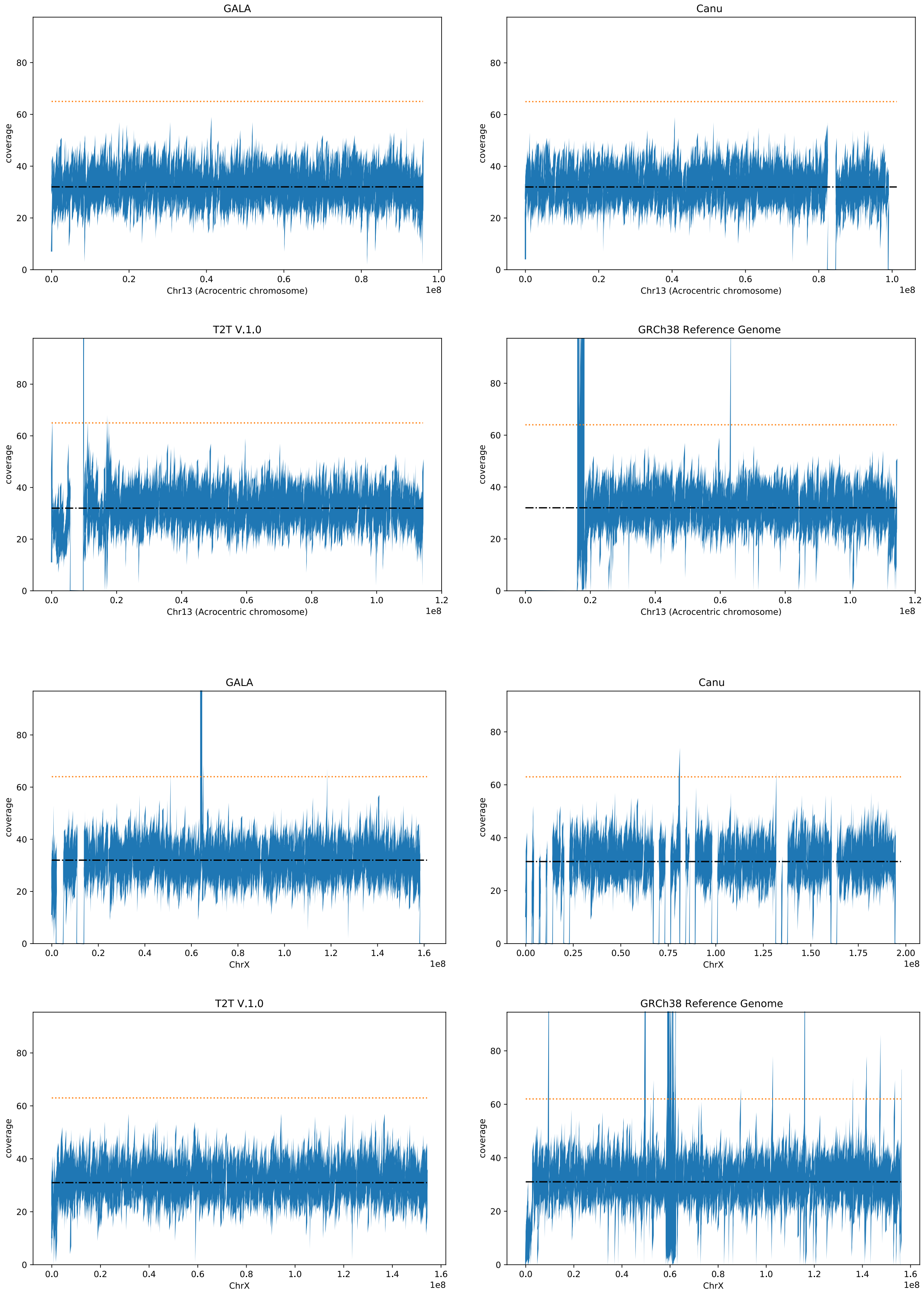

Supplementary Figure 7. Distributions of the depth-of-coverage when aligning the raw HiFi reads to the Gala assembly, final HiCanu assembly, T2T v1.0 assembly, GRCh38 human reference genome. For simplicity, only chr2, chr8, chr11, chr12, ch13, and chrX are shown here. The GALA assembly has very few gaps and shows comparable performance to the Canu assembly and the T2T v1.0 assembly. Note the final HiCanu assembly here is the one suggested by Nurk *et al* in [5] (by filtering out the contigs <50Kbp in "HiCanu 20kb HiFi" at https://obj.umiacs.umd.edu/marbl_publications/hicanu/chm13_20k_hicanu_hifi.fasta.gz).

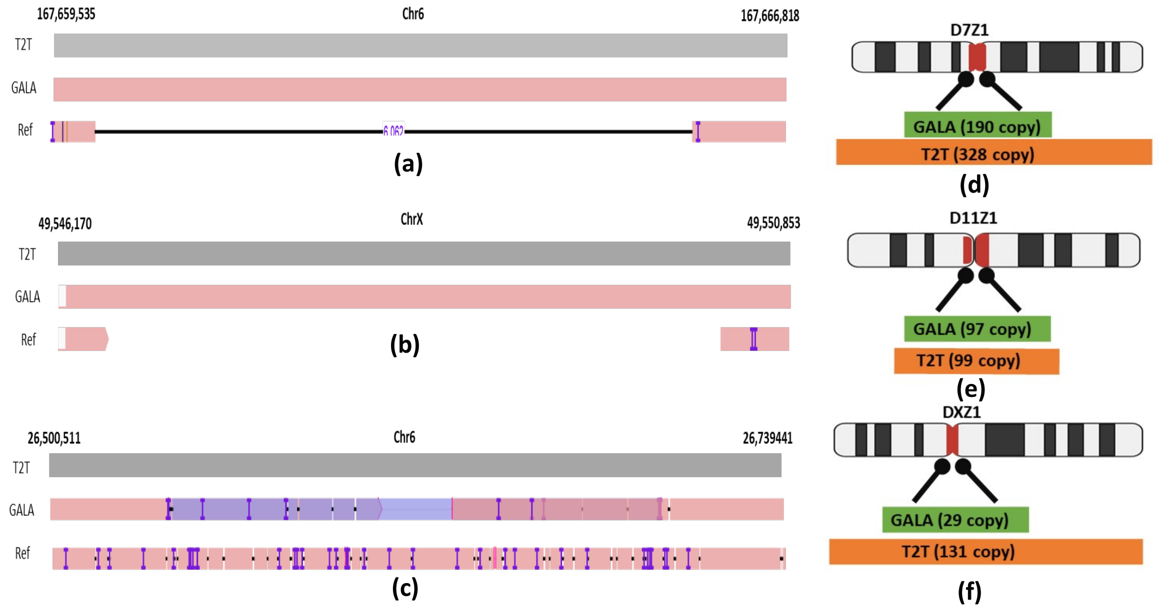

Supplementary Figure 8. (a, b) Two examples of gaps in the reference genome, which have been closed by GALA with 100 % identities compared to the T2T assembly. (c) The only one example in the human genome assembly in which the contig merging strategy has been applied. GALA successfully closed the gap by merging the overlapped fragment of the contigs. (d-f) Three repetitive regions where the GALA assembly and the T2T assembly have different number of copies, created with BioRender.com.

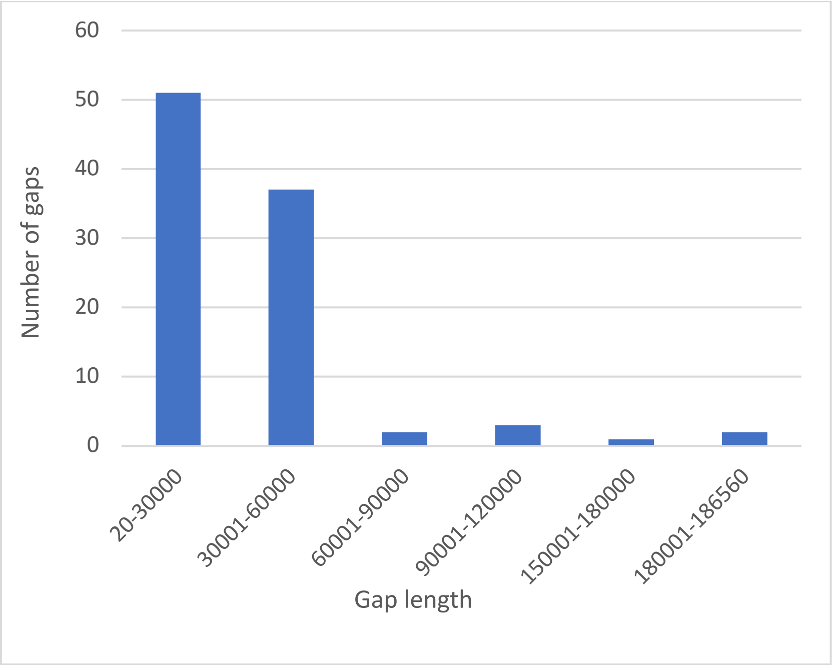

Supplementary Figure 9. The distribution of the length of gaps in the euchromatin regions in the human reference genome GRCH38.p13 which have been successfully closed by the GALA assembly when evaluated using the T2T assembly as the benchmark.

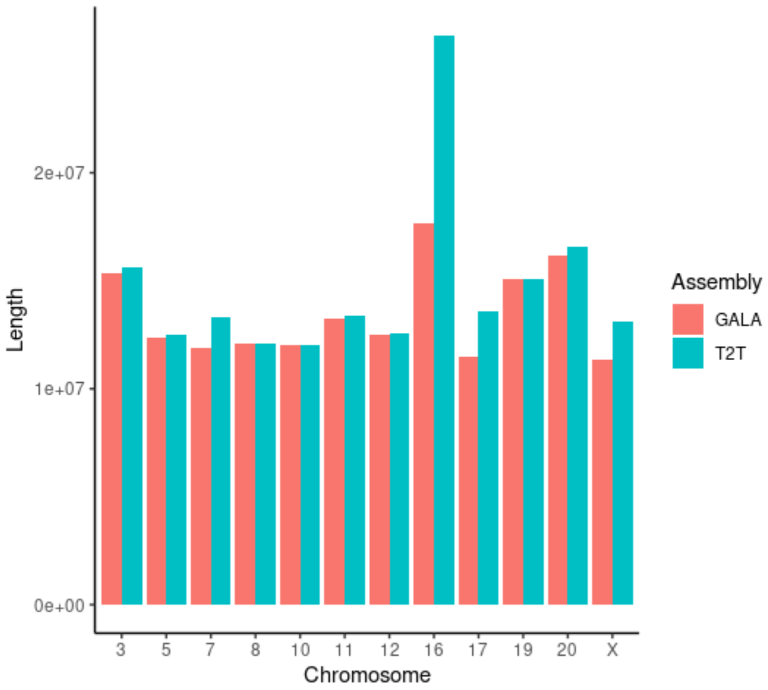

Supplementary Figure 10. The length of the centromeric repeats in the GALA assembly and the T2T assembly.

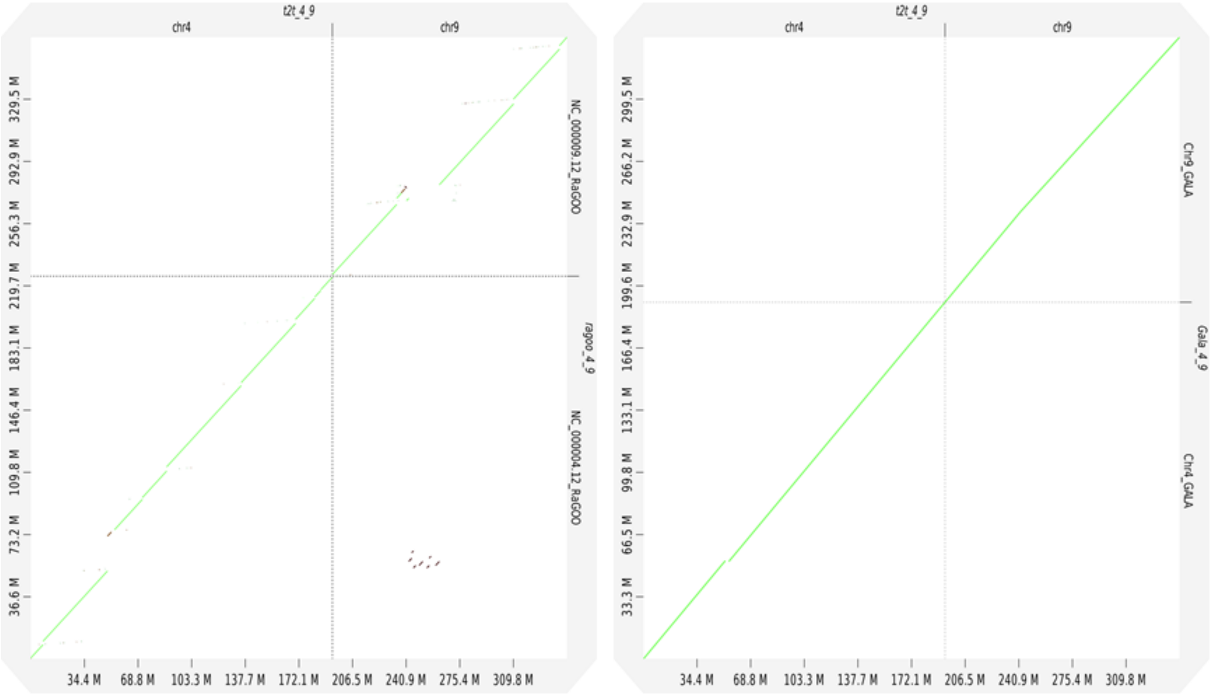

(a) (b)

Supplementary Figure 11. The dotter plot of alignments of (a) The reference-guided scaffolding and gap-filling of preliminary HiCanu assembly and (b) GALA assembly to the T2T assembly for chr4 and chr9.

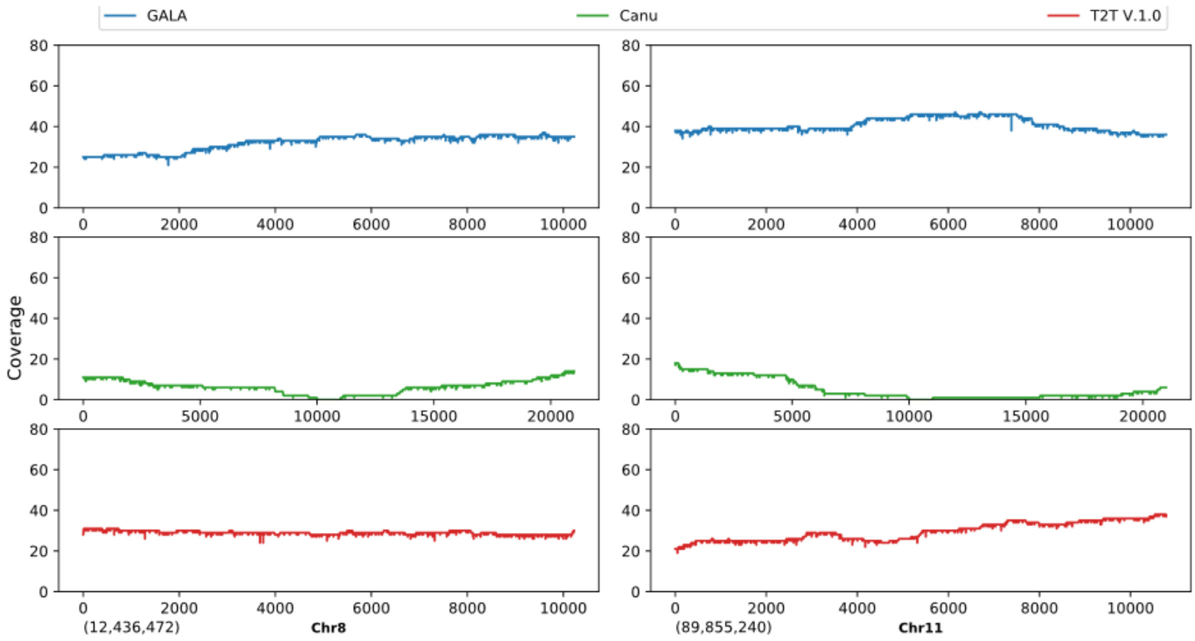

(a) **(b)**

Supplementary Figure 12. The coverage plot of the GALA assembly and the HiCanu assembly for two regions (a) in Chr8 and (b) in Chr11. Reads alignment to the GALA assembly, the HiCanu assembly and the T2T assembly indicated that GALA successfully assembled the two regions in chr8 and chr11 as good as the T2T assembly. The preliminary assembly of HiCanu contains duplicated sequences around the break points.

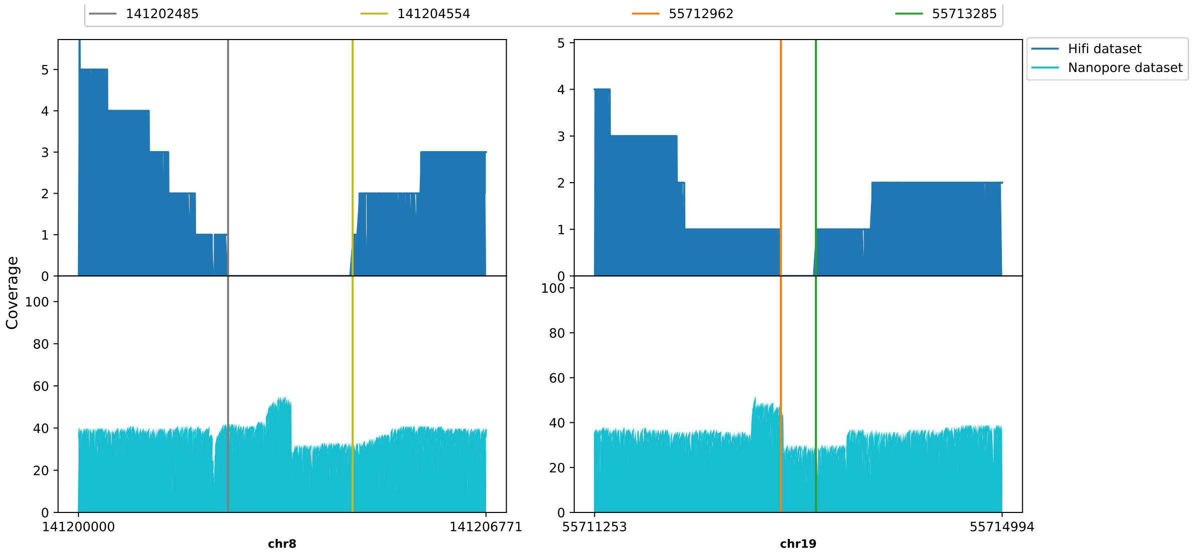

(a) **(b)**

Supplementary Figure 13. The coverage plot of two regions (a) in Chr8 and (b) in Chr19 where GALA failed to produce gap-free assembly. The upper subplots show the depth of raw HiFi reads, which are used by GALA for assembly, to the reference genome. Gaps indicate the missing of raw sequencing reads. The lower subplots show the depth of Nanopore reads from the same cell line (the sequencing data is from https://github.com/nanopore-wgs-consortium/CHM13 and is not used for our assembly), indicating the gaps are not caused by the divergence of the genome.

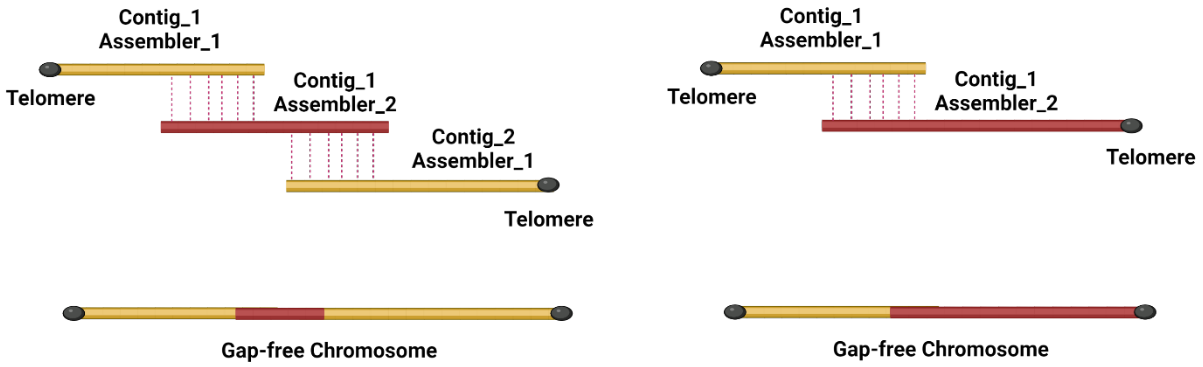

Supplementary Figure 14: The simple overlap-based strategy is used to merge contigs from different tools to achieve a gap-free chromosome. Minimap2 with "-x asm5" was used to do pairwise alignment among contigs assembled by Flye and miniasm. The contigs overlapped with each from one end were merged together.

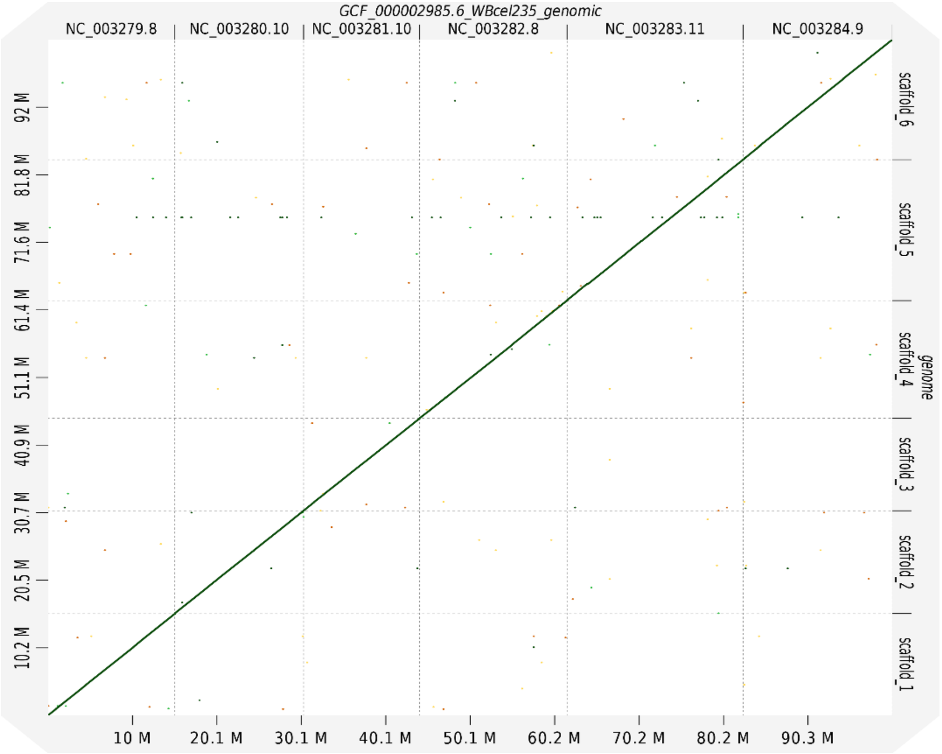

Supplementary Figure 15. Dotter plot of the alignments of our assembly to the *C. elegans* N2 reference genome using D-Genies [6].

Supplementary References

1. Koren, S. et al. Canu: scalable and accurate long-read assembly via adaptive k-mer weighting and repeat separation. *Genome Res* **27**, 722-736 (2017).
2. Li, H. & Durbin, R. Fast and accurate short read alignment with Burrows-Wheeler transform. *Bioinformatics* **25**, 1754-1760 (2009).
3. Gan, X. et al. Multiple reference genomes and transcriptomes for Arabidopsis thaliana. *Nature* **477**, 419-423 (2011).
4. Choi, J.Y. et al. Nanopore sequencing-based genome assembly and evolutionary genomics of circum-basmati rice. *Genome Biol* **21**, 21 (2020).
5. Nurk, S. et al. HiCanu: accurate assembly of segmental duplications, satellites, and allelic variants from high-fidelity long reads. *Genome Res* **30**, 1291-1305 (2020).
6. Cabanettes, F. & Klopp, C. D-GENIES: dot plot large genomes in an interactive, efficient and simple way. *PeerJ* **6**, e4958 (2018).
