# Supplementary material for: GALA: a computational framework for de novo chromosome-by-chromosome assembly with long reads
Source: Nat Commun. 2023 Jan 13;14:204. doi: 10.1038/s41467-022-35670-y (PMC9839709; doi:10.1038/s41467-022-35670-y)
Supplement: Supplementary file 3 — Reporting Summary [file 41467_2022_35670_MOESM3_ESM.pdf]

nature portfolio

Corresponding author(s): Xiangchao Gan
Last updated by author(s): Nov 29, 2022

Reporting Summary

Nature Portfolio wishes to improve the reproducibility of the work that we publish. This form provides structure for consistency and transparency in reporting. For further information on Nature Portfolio policies, see our Editorial Policies and the Editorial Policy Checklist.

Statistics

For all statistical analyses, confirm that the following items are present in the figure legend, table legend, main text, or Methods section.

n/a	Confirmed	
☐	☒	The exact sample size (*n*) for each experimental group/condition, given as a discrete number and unit of measurement
☐	☒	A statement on whether measurements were taken from distinct samples or whether the same sample was measured repeatedly
☐	☒	The statistical test(s) used AND whether they are one- or two-sided<br>*Only common tests should be described solely by name; describe more complex techniques in the Methods section.*
☒	☐	A description of all covariates tested
☒	☐	A description of any assumptions or corrections, such as tests of normality and adjustment for multiple comparisons
☐	☒	A full description of the statistical parameters including central tendency (e.g. means) or other basic estimates (e.g. regression coefficient) AND variation (e.g. standard deviation) or associated estimates of uncertainty (e.g. confidence intervals)
☒	☐	For null hypothesis testing, the test statistic (e.g. *F*, *t*, *r*) with confidence intervals, effect sizes, degrees of freedom and *P* value noted<br>*Give P values as exact values whenever suitable.*
☒	☐	For Bayesian analysis, information on the choice of priors and Markov chain Monte Carlo settings
☒	☐	For hierarchical and complex designs, identification of the appropriate level for tests and full reporting of outcomes
☒	☐	Estimates of effect sizes (e.g. Cohen's *d*, Pearson's *r*), indicating how they were calculated

Our web collection on statistics for biologists contains articles on many of the points above.

Software and code

Policy information about availability of computer code

Data collection	All data were downloaded using the direct download link and 'wget' command
Data analysis	The source code of GALA is available from github at https://github.com/ganlab/GALA under the MIT license. The version of the source code of GALA used in the study is available at https://doi.org/10.5281/zenodo.4674388. External software used in the current study were downloaded from the following URLs: assembly_stat.py commit daf6f29, https://github.com/mawad89/assembly_stats; Bcftools1.9, https://github.com/samtools/bcftools/releases; Busco 3.0.0, https://busco-archive.ezlab.org/v3; BWA 0.7.15-r1140, https://github.com/lh3/bwa; Canu 1.8,Canu 2.1 (Hicanu), https://github.com/marbl/canu; Fastq-sample b9a7f71 https://github.com/fplaza/fastq-sample; Flye 2.4, https://github.com/fenderglass/Flye; Hifiasm 0.5-dirty-r247, https://github.com/chhylp123/hifiasm; MECAT2 1.3, https://github.com/xiaochuanle/MECAT2; Miniasm 0.3-r179, https://github.com/lh3/miniasm; Minimap 0.2-r124-dirty https://github.com/lh3/minimap; Minimap2 2.17-r941, https://github.com/lh3/minimap2; NECAT 0.01, https://github.com/xiaochuanle/NECAT; PBJelly commit d4d2b10, https://github.com/esrice/PBJelly; pbmm2 1.1.0, https://github.com/PacificBiosciences/pbmm2; pilon 1.23, https://github.com/broadinstitute/pilon/releases; quiver 2.3.2, https://github.com/PacificBiosciences/GenomicConsensus; Racon 1.3.1, https://github.com/lbcb-sci/racon; Ragoo 1.1, https://github.com/malonge/RaGOO ;SALSA2 commit 974589f, https://github.com/marbl/SALSA; and Wtdbg2 2.5, https://github.com/ruanjue/wtdbg2.

For manuscripts utilizing custom algorithms or software that are central to the research but not yet described in published literature, software must be made available to editors and reviewers. We strongly encourage code deposition in a community repository (e.g. GitHub). See the Nature Portfolio guidelines for submitting code & software for further information.

Data

Policy information about availability of data

All manuscripts must include a data availability statement. This statement should provide the following information, where applicable:

- Accession codes, unique identifiers, or web links for publicly available datasets
- A description of any restrictions on data availability
- For clinical datasets or third party data, please ensure that the statement adheres to our policy

Genome assemblies generated by GALA in this study have been deposited in https://doi.org/10.5281/zenodo.6008862. The PacBio, Hi-C and Illumina reads of C. elegans used in this study are available at PRJNA430756 and the Nanopore sequencing data are available at PRJEB22098. Oryza sativa Dom Sufid Nanopore sequencing data are available at the European Nucleotide Archive (PRJEB32431). We downloaded the human dataset from https://obj.umiacs.umd.edu/marbl_publications/hicanu/index.html, https://obj.umiacs.umd.edu/marbl_publications/hicanu/chm13_20k_hicanu_hifi.fasta.gz. The C.elegans N2 reference genome is available at GCF_000002985.6. The VC2010 assembly is available at the European Nucleotide Archive (ENA; https://www.ebi.ac.uk/ena) under accession number PRJEB28388.

Human research participants

Policy information about studies involving human research participants and Sex and Gender in Research.

Reporting on sex and gender	Not applicable
Population characteristics	Not applicable
Recruitment	Not applicable
Ethics oversight	Not applicable

Note that full information on the approval of the study protocol must also be provided in the manuscript.

Field-specific reporting

Please select the one below that is the best fit for your research. If you are not sure, read the appropriate sections before making your selection.

☒ Life sciences ☐ Behavioural & social sciences ☐ Ecological, evolutionary & environmental sciences

For a reference copy of the document with all sections, see nature.com/documents/nr-reporting-summary-flat.pdf

Life sciences study design

All studies must disclose on these points even when the disclosure is negative.

Sample size	we descriped the sample size used in our analysis
Data exclusions	none
Replication	none
Randomization	none
Blinding	none

Reporting for specific materials, systems and methods

We require information from authors about some types of materials, experimental systems and methods used in many studies. Here, indicate whether each material, system or method listed is relevant to your study. If you are not sure if a list item applies to your research, read the appropriate section before selecting a response.

Materials & experimental systems

n/a	Involved in the study
☒	☐ Antibodies
☒	☐ Eukaryotic cell lines
☒	☐ Palaeontology and archaeology
☒	☐ Animals and other organisms
☒	☐ Clinical data
☒	☐ Dual use research of concern

Methods

n/a	Involved in the study
☒	☐ ChIP-seq
☒	☐ Flow cytometry
☒	☐ MRI-based neuroimaging
